# Honokiol inhibits ultraviolet radiation-induced immunosuppression through inhibition of ultraviolet-induced inflammation and DNA hypermethylation in mouse skin

**DOI:** 10.1038/s41598-017-01774-5

**Published:** 2017-05-10

**Authors:** Ram Prasad, Tripti Singh, Santosh K. Katiyar

**Affiliations:** 1 Department of Dermatology, University of Alabama at Birmingham, Birmingham, AL USA; 2 Birmingham Veterans Affairs Medical Center, Birmingham, AL USA; 3 Environmental Health Sciences, University of Alabama at Birmingham, Birmingham, AL USA; 4 Comprehensive Cancer Center, University of Alabama at Birmingham, Birmingham, AL USA

## Abstract

Ultraviolet (UV) radiation exposure induces immunosuppression, which contributes to the development of cutaneous malignancies. We investigated the effects of honokiol, a phytochemical found in plants of the genus *Magnolia*, on UVB-induced immunosuppression using contact hypersensitivity (CHS) as a model in C3H/HeN mice. Topical application of honokiol (0.5 and 1.0 mg/cm^2^ skin area) had a significant preventive effect on UVB-induced suppression of the CHS response. The inflammatory mediators, COX-2 and PGE_2_, played a key role in this effect, as indicated by honokiol inhibition of cyclooxygenase-2 (COX-2) expression and PGE_2_ production in the UVB-exposed skin. Honokiol application also inhibited UVB-induced DNA hypermethylation and its elevation of the levels of TET enzyme, which is responsible for DNA demethylation in UVB-exposed skin. This was consistent with the restoration of the CHS response in mice treated with the DNA demethylating agent, 5-aza-2′-deoxycytidine, after UVB exposure. There was no significant difference in the levels of inhibition of UVB-induced immunosuppression amongst mice that were treated topically with available anti-cancer drugs (imiquimod and 5-fluorouracil). This study is the first to show that honokiol has the ability to inhibit UVB-induced immunosuppression in preclinical model and, thus, has potential for use as a chemopreventive strategy for UVB radiation-induced malignancies.

## Introduction

Constant exposure of the skin to solar ultraviolet (UV) radiation induces a variety of harmful effects, including premature aging or photoaging of the skin as well as a heightened risk of skin cancers (both melanoma and non-melanoma)^1–3^. The immunosuppressive effects of UV radiation, particularly in the UVB spectrum (290–320 nm wavelengths), are considered to represent one of the most important environmental risk factors for development of skin cancers in humans^1–3^. This concept is supported by the observation that chronically immunosuppressed patients living in regions of intense sun exposure experience a higher rate of cutaneous malignancies^4^. The incidence of skin cancers is also high among organ transplant recipients who receive continuous immunosuppressive therapy^5^. Considerable experimental data indicate that UVB radiation-induced suppression of the immune system contributes to the development of skin tumors^6, 7^.

UV-induced inflammation is considered to be an early event in skin tumor promotion and progression. UV-induced inflammatory responses result in the development of erythema, edema, and hyperplastic responses, as well as increases in the levels of cyclooxygenase-2 (COX-2) and prostaglandin (PG) metabolites. The UV-induced PG metabolites (PGE_2_, PGD2 and PGF_2α_) have been implicated in UVB-induced immunosuppression^8^. Several studies show that nonsteroidal anti-inflammatory drugs (NSAIDs), which exert their anti-inflammatory and anti-tumor promoting effects through inhibition of COX-2 and PGs, can reverse the immunosuppressive effects of UV radiation^9, 10^. Moreover, it has been reported that the use of NSAIDs reduced the risk of skin cancer^11^. Among the PG metabolites, PGE_2_ is the major and most reactive metabolite and considered to be a potent mediator of inflammatory reactions. Studies have suggested that PGE_2_ plays a key role in UV-induced immunosuppression and epidemiological and clinical data as well as studies in laboratory animals suggest a link between inflammation, immunosuppression and skin cancer^3, 8^. We have detected a distinct pattern of DNA hypermethylation in UVB-exposed mouse skin as well as in UVB-induced skin tumors in mice and this pattern is linked to the inflammation in UV exposed skin^12^. Collectively, these data suggest that PGE_2_ promotes UVB-induced immunosuppression and that the PGE_2_ may act to suppress the immune reactivity by promoting DNA methylation in UVB-exposed skin^13, 14^.

As greater than 3 million new cases of skin cancers are diagnosed each year in the United States, this disease represents a major public health problem. It has been estimated that the cost of treating melanoma and non-melanoma skin cancers in the United States is approximately $3.0 billion annually^1, 2^. Although some drugs are approved for the treatment of skin cancer, their usefulness is limited by toxicities and the development of resistance over time. Thus, new and promising strategies are urgently needed to alleviate the burden of this major public health problem. In previous studies, we have shown that topical application of honokiol, a phytochemical found in plants of the genus *Magnolia*, significantly inhibits UVB-induced skin tumor development in terms of tumor multiplicity and tumor growth/size. Additionally, honokiol has the ability to prevent the transformation of papillomas to carcinomas^15^. The anti-tumor effects of honokiol were associated with a decrease in inflammatory responses and cell cycle regulatory proteins^15–18^. However, the exact mechanism and/or molecular targets of the anti-skin cancer effects of honokiol are unclear. As, UVB-induced immunosuppression has been implicated in skin cancer risk, we assessed the effects of honokiol on UVB-induced suppression of the immune system. For this purpose, we used a mouse contact hypersensitivity (CHS) model, which is considered to be a prototype of T-cell mediated immune response^1–3, 19^.

## Results

### Honokiol inhibits UVB-induced suppression of the CHS response in mice

**Figure 1:**
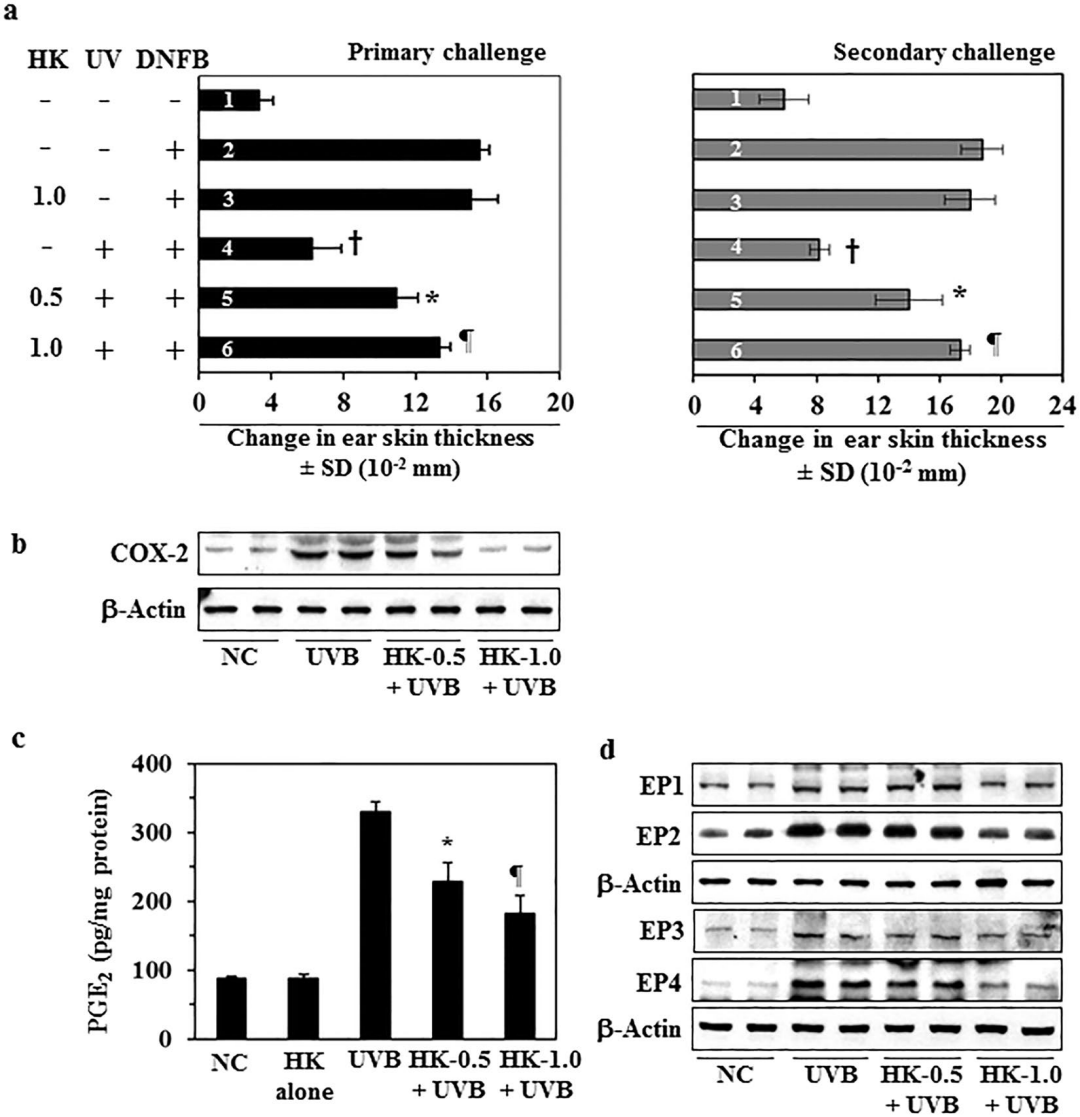
Topical application of honokiol inhibits UVB-induced suppression of the CHS response in mice through inhibition of inflammatory mediators. The clipper-shaved dorsal skin of female C3H/HeN mice was exposed to UVB radiation (150 mJ/cm^2^) for four consecutive days. Honokiol (0.5 and 1.0 mg/cm^2^ of skin area) was applied in a hydrophilic cream-based topical formulation before each exposure. (**a**, left panel) The mice were then sensitized to DNFB, and the CHS response to application of DNFB on the ear skin (challenge) was assessed by measurement of the ear swelling 24 h later. The change in ear skin thickness (swelling) is reported in millimeter (mm × 10^−2^) as the mean ± SD, n = 4–5 per group. (**a**, right panel), Long-term effects of honokiol tested by secondary challenge. Significant inhibition *versus* positive control group, ^†^ *P* < 0.001, significant increase in CHS response *versus* non-honokiol treated and UVB-irradiated mice, ^*^ *P* < 0.01; ^¶^ *P* < 0.001. (**b**–**d**) The mice were exposed to UVB (150 mJ/cm^2^) radiation on four consecutive days with and without topical application of honokiol and sacrificed 24 h after the last UVB exposure. Dorsal skin samples from the mice were collected for analysis. (**b**) Topical application of honokiol inhibits UVB-induced COX-2 expression in mouse skin. COX-2 levels were determined using western blot analysis. (**c**) Topical application of honokiol inhibits the UVB-induced increase in the expression levels of PGE_2_. The concentration of PGE_2_ in skin homogenates was determined using a PGE_2_ immunoassay kit. PGE_2_ concentration is expressed in terms of pg/mg protein as the mean ± SD. Significant inhibition *versus* non-honokiol-treated control group, ^*^ *P* < 0.01, ^¶^ *P* < 0.001. (**d**) The levels of the PGE_2_ receptors (EP1, EP2, EP3, and EP4) were determined in skin samples using western blot analysis. For panel b and d, western blot data are shown. Samples was prepared by pooling the skin biopsies from at least two mice in each treatment group. NC, normal control group.

As topical application of honokiol prevents UVB-induced skin tumor growth and multiplicity in laboratory animals^15, 16^, we tested whether treatment of mice with honokiol in a hydrophilic topical formulation protects against UVB-induced suppression of the CHS response to the skin contact sensitizer, 2, 4-dinitrofluorobenzene (DNFB). Topical application of honokiol (1.0 mg/cm^2^ skin area) did not affect the ability of the mice to generate a CHS response to DNFB in the absence of UVB irradiation (Fig. 1a, left panel, compare the CHS response of the third bar with the second bar, *i.e*. positive control). In the absence of treatment with honokiol, the CHS response in terms of ear swelling was significantly lower (75% suppression, *P* < 0.001; 4^th^ bar) in those mice that were UVB-irradiated than those mice that were not UVB-irradiated (left panel, 2^nd^ bar, positive control), confirming the immunosuppressive effect of the UVB radiation in these mice. The UVB-induced suppression of CHS was significantly lower in the groups of mice that were treated with honokiol at a concentration of either 0.5 or 1.0 mg/cm^2^ skin area (5^th^ and 6^th^ bar) prior to UVB irradiation (38% and 57% lower; *P* < 0.01 to *P* < 0.001) than in the UVB-irradiated mice that were not treated with honokiol (Fig. 1a, left panel, 4^th^ bar). Topical treatment with a lower concentration of honokiol (0.2 mg/cm^2^ skin area) failed to provide significant protection from UVB-induced suppression of the CHS response in these mice (data not shown). These results demonstrated that topical treatment with honokiol in a hydrophilic topical formulation at doses of honokiol of 0.5 and 1.0 mg/cm^2^ skin area of the mice is capable of protecting mice from UVB-induced immunosuppression.

To determine whether the topical application of honokiol stimulates long-term effects in the UVB-exposed mice, the mice from the previous experiment (Fig. 1a, left panel, primary challenge) were rested for 6 weeks after primary challenge with DNFB. The mice were not treated with honokiol and were not exposed to UVB during this time period. The mice were then rechallenged with DNFB. As shown in Fig. 1a (right panel), the groups of mice that had been topically treated with honokiol at a concentration of 0.5 or 1.0 mg/cm^2^ skin area 6 weeks prior to the rechallenge exhibited a significantly greater CHS response (5^th^ and 6^th^ bar, 45–71% more, *P* < 0.001) after rechallenge with DNFB (secondary challenge, right panel) than the UVB-irradiated mice that had not been treated with honokiol at any stage (4^th^ bar). These observations suggest that the ability of honokiol to protect the mice from UVB-induced immunosuppression persists for some time after its topical application.

### Honokiol-mediated protection of the immune system in UVB-irradiated mice is associated with suppression of the levels of inflammatory mediators

It has been shown that UVB-induced suppression of CHS response is associated with an increase in the levels of inflammatory mediators, such as COX-2 expression and PGE_2_ production, in UVB-exposed skin^6^. Our previous studies show that inhibition of UVB-induced suppression of CHS is mediated primarily through inhibition of COX-2 expression in UVB-irradiated mice^8, 14^. As topical treatment of honokiol inhibited UVB-induced suppression of the CHS response (Fig. 1a), we assessed whether inhibition of UVB-induced suppression of CHS by honokiol is associated with a reduction in the levels of inflammatory mediators. For this purpose, C3H/HeN mice were exposed to UVB radiation (150 mJ/cm^2^) on four consecutive days with and without topical treatment of honokiol (0.5 and 1.0 mg/cm^2^) 30 min prior to each UVB exposure. Twenty-four hours after the last UVB exposure, mice were sacrificed and skin samples collected. Lysates of the skin samples were prepared and subjected to western blot analysis. Western blot analysis confirmed higher levels of COX-2 expression in UVB-exposed skin than non-UVB exposed control mouse skin and topical treatment with honokiol reduced the UVB-induced increase in COX-2 expression in mouse skin in a dose-dependent manner (Fig. 1b).

PGE_2_, which is considered to be potent mediator of inflammation, has been implicated in UVB-induced immunosuppression^3, 8, 14^. As shown in Fig. 1c, honokiol treatment significantly decreased the production of PGE_2_ (42% to 61%, *P* < 0.001) in UVB-exposed mouse skin. PGE_2_ exerts its effects primarily through its receptors (EP1, EP2, EP3, and EP4). The levels of EP1, EP2, EP3 and EP4 in UVB-exposed skin were higher than the levels in non-UVB-exposed normal skin. Honokiol treatment markedly reduced the levels of the EP1, EP2 and EP4 receptors in UVB-exposed skin as compared to non-honokiol-treated but UVB-irradiated mouse skin (Fig. 1d). These findings indicate that the inhibitory effect of honokiol on UVB-induced immunosuppression is mediated, at least in part, through its inhibitory effects on inflammatory mediators.

### Honokiol does not inhibit UVB-induced suppression of CHS in COX-2-deficient mice

**Figure 2:**
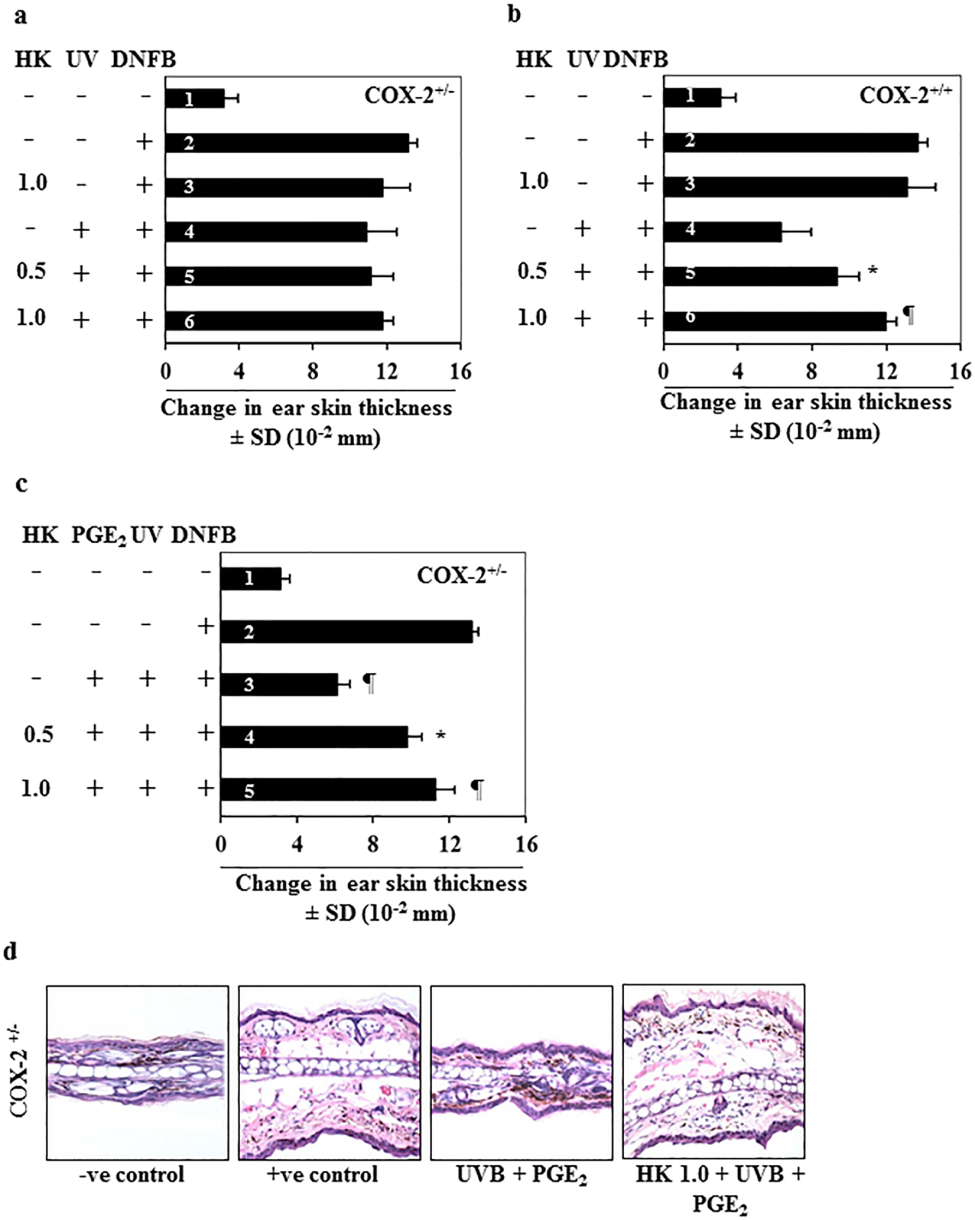
Honokiol does not inhibit UVB-induced suppression of CHS response in COX-2-deficient mice. COX-2-deficient mice were treated and analyzed as described in Fig. 1(a and b **)**. Topical application of honokiol does not prevent UVB-induced suppression of CHS response in COX-2-deficient mice. COX-2-deficient mice were resistant to UVB-induced suppression of the CHS response. (**b**) The wild-type littermates of COX-2-deficient mice show UVB-induced suppression of CHS, and topical application of honokiol inhibits UVB-induced suppression of CHS in these mice. Significant suppression of the CHS response *versus* UVB exposure in the absence of honokiol treatment. ^*^ *P* < 0.01, ^¶^ *P* < 0.001. (**c**) Topical treatment of PGE_2_ after each UVB exposure promotes suppression of CHS in UVB-exposed COX-2-deficient mice (3^rd^ bar), and topical application of the mice with honokiol prior to the UVB exposure (4^th^ and 5^th^ bar) stimulates the CHS response in the PGE_2_ treated and UVB-irradiated COX-2-deficient mice. Significant suppression of CHS (3^rd^ bar) *versus* positive control group (2^rd^ bar), ^¶^P < 0.001. Significant restoration of CHS response by honokiol *versus* PGE_2_ induced suppression of CHS, ^*^ *P* < 0.01, ^¶^ *P* < 0.001. (**d**) Histologic (H&E staining) evaluation of ear skin sections obtained from COX-2-deficient (COX-2^+/−^) mice with and without treatment with PGE_2_ and honokiol and subjected to the CHS protocol. Topical administration of PGE_2_ to UVB-exposed COX-2-deficient mice resulted in reduced ear skin thickness as compared to the positive (+ve) control group of mice that were sensitized with DNFB, while topical application of honokiol prior to UVB+PGE_2_ treatment enhanced the thickness of the ear skin and infiltration of leukocytes in the ear skin. Representative micrographs are shown from mice, n = 4.

To further determine the association of the inhibitory effects of honokiol on UVB-induced immunosuppression and its effects on COX-2 expression, we used COX-2-deficient mice. As shown in Fig. 2a, the sensitization reactions after DNFB challenge (4^th^ bar) in UVB-exposed COX-2 deficient mice were not significantly different from the positive control groups (2^nd^ and 3^rd^ bars). Topical application of honokiol did not significantly affect the CHS response in the UVB-irradiated COX-2-deficient mice (5^th^ and 6^th^ bar) compared with non-honokiol-treated and UVB-exposed COX-2-deficient mice (4^th^ bar). In contrast, topical treatment with honokiol significantly inhibited UVB-induced suppression of CHS (53%, *P* < 0.001) in the wild-type littermates of the COX-2-deficient mice (Fig. 2b). These observations indicate that COX-2 expression is required for UVB-induced immunosuppression and honokiol inhibits UVB-induced immunosuppression through its inhibitory effects on COX-2 upregulation.

As PGE_2_ is a major metabolite of COX-2 and mediates COX-2 effects, we further determined the effects of honokiol on UVB-induced suppression of CHS by analysis of the CHS responses in COX-2-deficient mice after treatment with PGE_2_. We observed that COX-2-deficient mice that were UVB irradiated and treated topically with PGE_2_ showed significant suppression (>70%, *P* < 0.001) of the CHS response (Fig. 2c, 3^rd^ bar). Treatment with honokiol inhibited this PGE_2_-mediated suppression of the CHS response (37% to 52%, *P* < 0.01 to *P* < 0.001) in UVB-irradiated mice as compared with mice that were treated with PGE_2_ and exposed to UVB but not treated with honokiol (Fig. 2c). The skin swelling response in CHS is a reflection of the massive leukocyte infiltration. Histologic examination of the ear skin swelling responses in the different treatment groups (Fig. 2d), confirmed enhanced ear skin thickness and higher cell numbers in PGE_2_-treated and UVB-irradiated COX-2-deficient mice as compared to non-honokiol-treated but PGE_2_-treated UVB exposed COX-2-deficient mice (Fig. 2d).

### Honokiol prevents UVB-induced DNA hypermethylation in mouse skin

**Figure 3:**
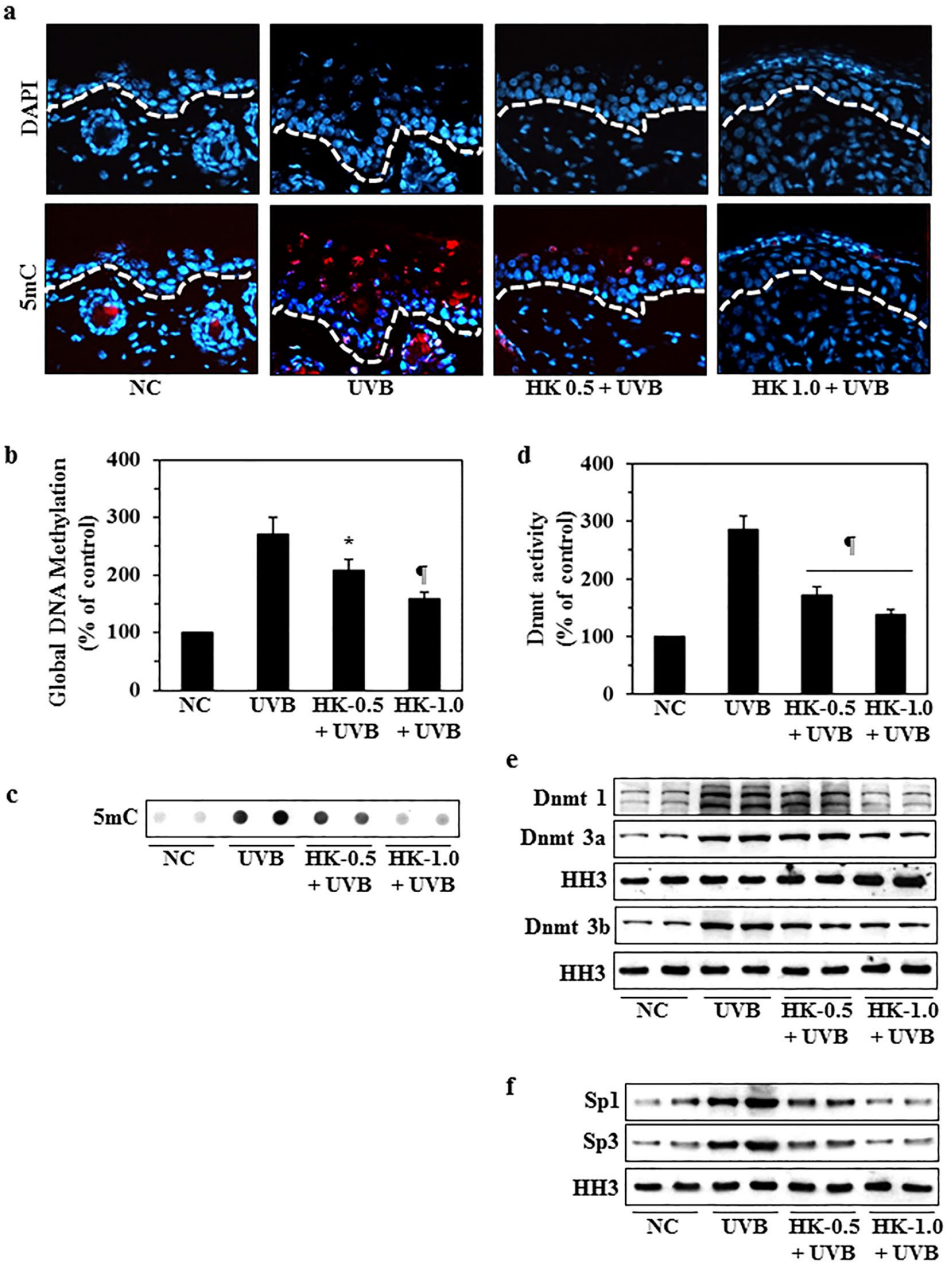
Topical application of honokiol inhibits UVB-induced DNA hypermethylation and DNA methyltransferase activity in UVB-irradiated mouse skin. Clipper-shaved mouse skin was exposed to UVB radiation (150 mJ/cm^2^) for 4 consecutive days with and without topical application of honokiol. Mice were sacrificed 24 h after the last UVB exposure. Skin samples from each mouse were collected for analysis. (**a**) Representative micrographs of skin samples subjected to immunohistochemical detection of DNA methylation-positive (5mC^+^) cells using a 5mC-specific antibody. 5mC^+^ cells appear red. White line represent the boundary between epidermis and dermis. (**b**) Epidermal DNA was extracted and subjected to analysis of global DNA methylation using a Global DNA Methylation Kit. (**c**) The levels of 5-mC were estimated in DNA samples from different groups by dot-blot analysis. A representative dot-blot is shown. (**d**) Nuclear extracts were prepared and total Dnmt activity determined using the Dnmt Activity Assay Kit. Data are presented in terms of percentage *versus* non-UVB-exposed control skin samples. Significant inhibition *versus* the group exposed to UVB but not treated with honokiol, ^*^ *P* < 0.01, ^¶^ *P* < 0.001. (**e**) The skin samples from different treatment groups were also analyzed for the levels of Dnmt1, Dnmt3a and Dnmt3b proteins using western blot analysis. (**f**) The expression levels of the transcription factors, Sp1 and Sp3, were determined by western blotting. Equal nuclear protein loading on gels was verified using anti-histone H3 antibody (HH3).

Previously, we showed that UVB irradiation induces DNA hypermethylation and increases DNA methyl transferase (Dnmt) activity in UVB-exposed skin and UVB-induced skin tumors^12, 13^. The DNA hypermethylation pattern in UVB-exposed mouse skin was associated with the increased levels of inflammatory mediators, including COX-2 overexpression and elevated levels of PGE_2_ production. We therefore further investigated the effects of honokiol on UVB-induced epigenetic regulators using the C3H/HeN mouse model and a treatment protocol identical to that described in Fig. 1 for the analysis of expression of COX-2 and PGE_2_ except that the skin samples were analyzed for epigenetic regulators. As shown in Fig. 3a, higher numbers of 5-methyl cytosine (5-mC)-positive cells were detected by immunofluorescence analysis in UVB-irradiated mouse skin than in non-UVB-exposed normal skin. Topical application of honokiol inhibited this UVB-induced formation of 5-mC-positive cells in a dose-dependent manner. These observations were verified through analysis of the levels of global DNA methylation levels in skin samples from the different treatment groups using the global DNA Methylation Quantification Kit (Fig. 3b). Topical application of honokiol significantly inhibited (37–65%, *P* < 0.01 to *P* < 0.001) the levels of global DNA methylation in UVB-exposed mouse skin as compared to non-honokiol-treated UVB-exposed skin. Futhermore, the results of dot-blot analysis using an antibody specific for 5-mC to test the levels of 5-mC in lysates of skin samples were consistent with the findings described above (Fig. 3c).

Dnmt is the key regulatory enzyme in the DNA methylation pathway^13^. On examination of the effects of honokiol on Dnmt activity, we found that topical application of honokiol significantly inhibited the UVB-induced increase in the levels of Dnmt activity by 61–79% (*P* < 0.001) (Fig. 3d) and simultaneously reduced the expression of the Dnmt1, Dnmt3a and Dnmt3b proteins (Fig. 3e). It is known that Dnmts are transcriptionally regulated by the combinational actions of proteins that bind to distinct promoter and enhancer elements, including the transcription factors Sp1 and Sp3^20, 21^. We found that the expression levels of both Sp1 and Sp3 were enhanced after UVB exposure of the skin and that this UVB-induced increase in the expression of these transcription factors was inhibited by topical application of honokiol to the skin prior to UVB exposure (Fig. 3f).

### Honokiol stimulates TET enzyme (methylcytosine dioxygenase) activity and elevates the levels of TET proteins in UVB-exposed mouse skin

**Figure 4:**
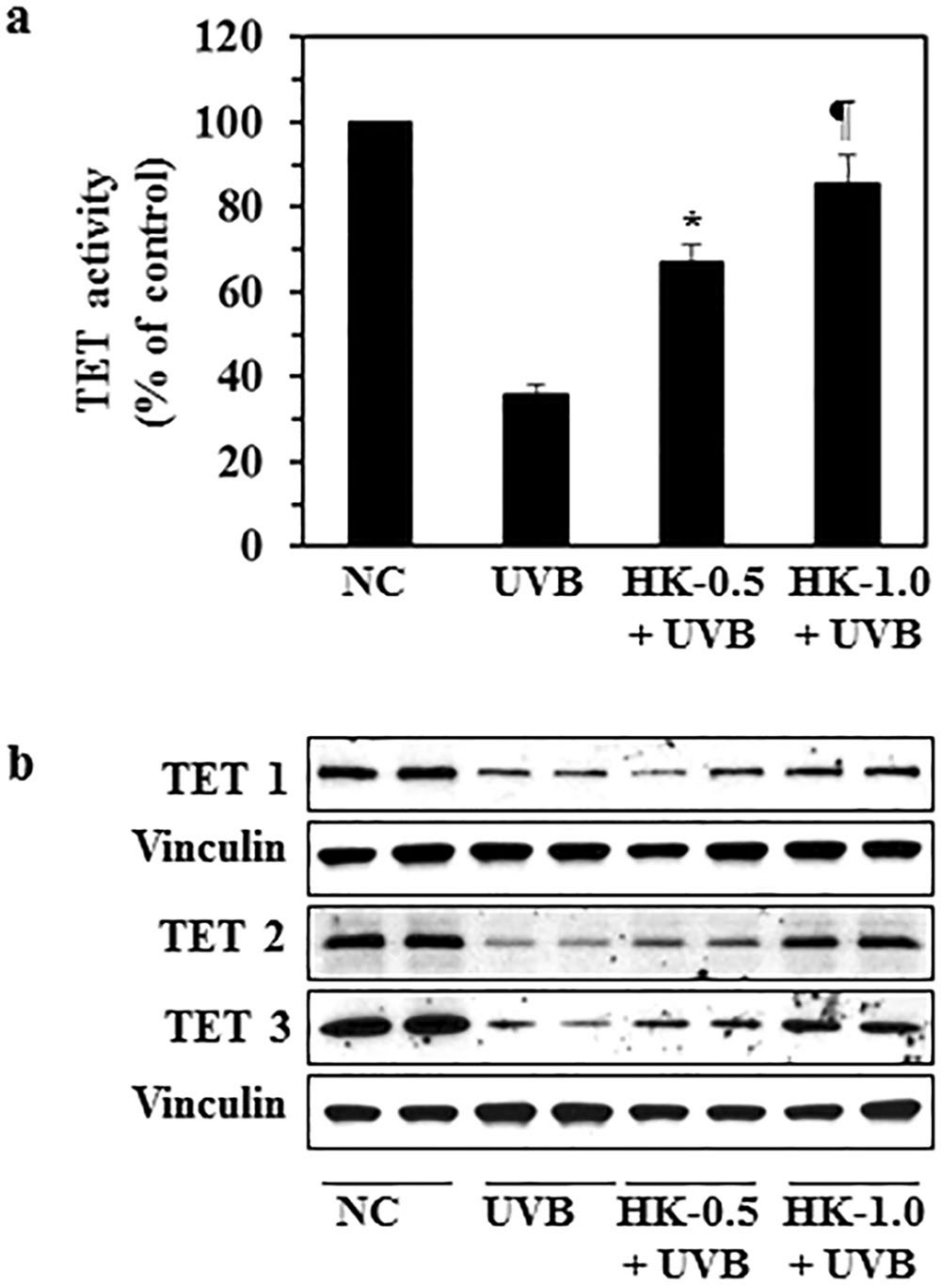
Topical application of honokiol restores the levels of TET enzyme activity as well as TET proteins in UVB-exposed mouse skin. Mice were exposed to UVB radiation (150 mJ/cm^2^) for 4 consecutive days with and without topical application of honokiol. Mice were sacrificed 24 h after the last UVB exposure, n = 4/group. (**a**) Skin lysates were subjected to the analysis of TET activity using the TET Activity Assay Kit and data are presented in terms of percent of control (non-UVB-irradiated control skin). Significant increase *versus* the group exposed to UVB but not treated with honokiol, ^*^ *P* < 0.01, ^¶^ *P* < 0.001. (**b**) The skin lysate samples were analyzed for levels of TET 1, TET 2, and TET 3 proteins using western blot analysis. Equal protein loading on gels was verified using anti-vinculin antibody. Each sample was prepared by pooling the skin biopsies from at least two mice in each treatment group. NC, normal control group.

The abnormal pattern of DNA methylation at cytosine bases in the genome has been studied in several diseases including skin cancer^12, 13^ and is tightly linked to gene expression. It has been shown that the ten eleven translocation (TET) enzymes that catalyze demethylation of 5-methyl cytosine (5-mC) and promote locus-specific reversal of DNA hypermethylation^22^. We therefore determined the effect of honokiol on the activity of the TET enzymes and the levels of TET proteins in UVB-exposed mouse skin. As shown in Fig. 4a, UVB irradiation significantly downregulated TET activity in UVB-exposed mouse skin as compared to non-UVB-exposed normal mouse skin. Topical application of honokiol significantly restored TET activity (50–77%, *P* < 0.01, *P* < 0.001) in UVB-exposed skin. Topical application of honokiol also reactivated or restored the levels of TET proteins (TET1, TET2 and TET3) in the UVB-exposed mouse skin as compared to non-honokiol-treated UVB-exposed mouse skin (Fig. 4b). These observations suggest that the honokiol-mediated reduction in UVB-induced DNA hypermethylation is associated with an activation of TET enzyme and restoration of TET protein expression in the skin.

### A DNA demethylating agent and a COX-2 inhibitor stimulate the CHS response in UVB- exposed mice

**Figure 5:**
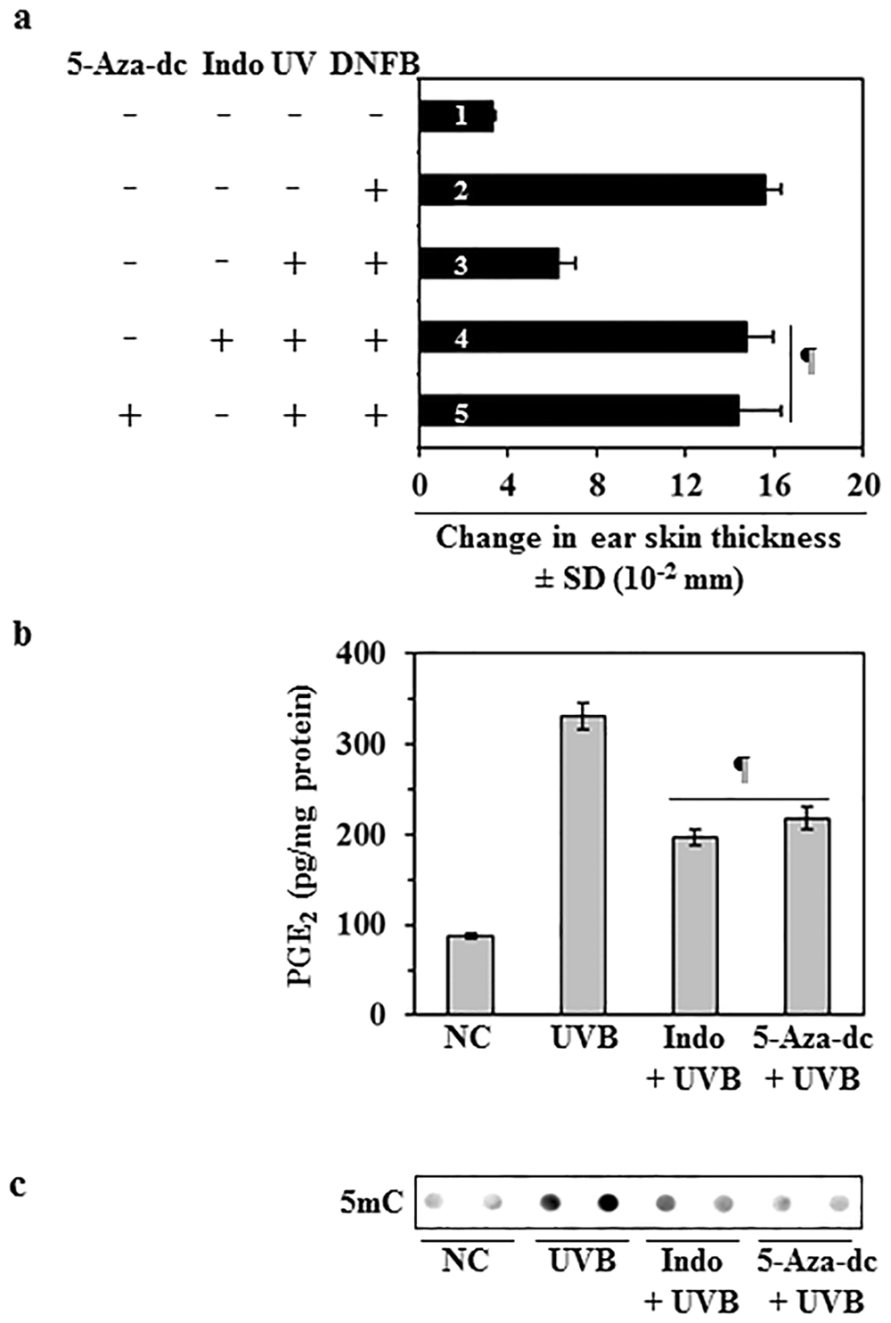
Treatment with a DNA demethylating agent or COX-2 inhibitor enhances the CHS response in UVB-exposed mice. (**a**) Treatment with indomethacin (a COX-2 inhibitor) or 5-Aza-dc (a DNA demethylating agent) enhances CHS response in UVB-exposed mice. Mice were UVB exposed and sensitized to DNFB as detailed in Fig. 1. The change in ear skin thickness is presented in millimeter (mm × 10^−2^) as the mean ± SD, n = 4 per group. Mice in group 1 were not sensitized with DNFB but only challenged with DNFB on ear skin and serve as a negative control. Mice in group 2 to 5 were sensitized and challenged with DNFB. Mice in group 4 were treated with indomethacin and mice in group 5 were treated with 5-Aza-dc (1.0 mg/kg body weight, i. p.) by topical application 30 min before each UVB irradiation. Significant difference in CHS response *versus* UVB exposure alone in the absence of indomethacin and 5-Aza-dc treatment (bar/group #3), ^¶^ *P* < 0.001. (**b**) The levels of PGE_2_ in the dorsal skin samples were determined using a PGE_2_ Immunoassay Kit. Data are shown as the means ± SD. Significant inhibition *versus* UVB alone exposed group, ^¶^ *P* < 0.001. **(c)** Skin DNA was subjected to the analysis of 5-mC using dot-blot analysis.

Collectively, the data presented above indicate that inhibition of UVB-induced suppression of CHS by topical application of honokiol is mediated through inhibition of inflammatory mediators and DNA demethylation in UVB-exposed skin. To test whether inhibition of inflammatory mediators and DNA demethylation can inhibit UVB-induced suppression of CHS, we treated C3H/HeN mice with a known COX-2 inhibitor, indomethacin, and a known DNA demethylating agent, 5-aza-2′-deoxycytidine (5 Aza-dc (a), using the protocol described in the Materials and Methods section. As shown in Fig. 5a, treatment of mice with indomethacin (4^th^ bar) and 5 Aza-dc (5^th^ bar) significantly inhibited (66% to 69%, *P* < 0.001) the UVB-induced suppression of the CHS response in mice (4^th^ and 5^th^ bar) as compared to the control group (3^rd^ bar). We also found that the production of PGE_2_ was significantly lower in both the indomethacin (55%, *P* < 0.001)-treated and the 5Aza-dc (46%, *P* < 0.001)-treated UVB-exposed mouse skin as compared to UVB-exposed mouse skin that was not treated with these agents (Fig. 5b). Further, the treatment of indomethacin and 5-Aza-dc also inhibited the levels of DNA methylation in UVB-exposed skin as observed by dot blot analysis in which the levels of DNA methylation were determined using an antibody specific for 5-mC (Fig. 5c).

### Effect of honokiol treatment (topical *vs* oral administration) on UVB-induced immunosuppression

**Figure 6:**
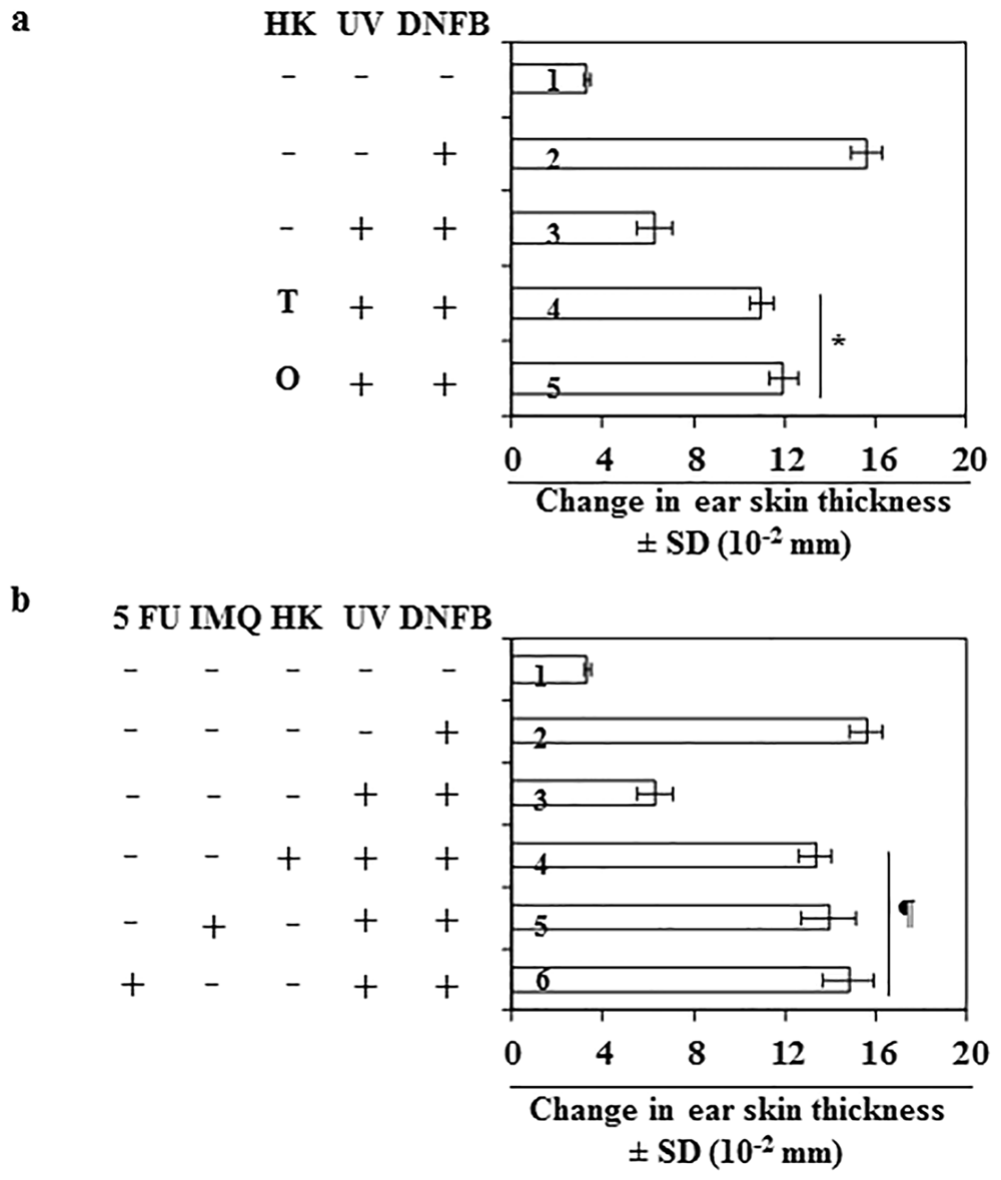
Comparative effect of honokiol (topical vs oral) and other anti-skin cancer drugs on UVB-induced suppression of the CHS response in C3H/HeN mice. The CHS protocol was followed as detailed under Fig. 1, n = 5/group. (**a**) Mice in group-4 were administered honokiol by topical application (2.0 mg/mouse) and the mice in group-5 were administered honokiol by oral gavage (2.0 mg/mouse) 30 min before each UVB irradiation. The CHS response was measured. Significant increase in CHS response (groups 4 and 5) *versus* UVB exposure in the absence of honokiol treatment (group-3). *P < 0.001. T, topical treatment of honokiol; O, treatment of honokiol by oral gavage. (**b**) Comparative effects of equimolar concentrations of honokiol, imiquimod and 5-fluorouracil on the UVB-induced suppression of the CHS response. The CHS protocol used was identical to that described for panel (**a**). Mice in groups 4, 5 and 6 were treated topically with honokiol, imiquimod or 5-fluorouracil in equimolar concentration (18.8 mM) 30 min before each UVB exposure. Significant difference in CHS response *versus* UVB exposure in the absence of any agent treatment (group-3), ^¶^ *P* < 0.001, n = 4 per group.

Potentially, honokiol could be administered in an oral form or applied topically. Each route of administration has advantages and disadvantages. Therefore, we compared the effects of topical application and oral administration of honokiol on UVB-induced immunosuppression in mice using the CHS model. In this set of experiments, honokiol was administered by topical application (2 mg/mouse; equivalent to 100 mg/kg body weight) or by oral gavage (2 mg/mouse). Treatment with honokiol either by topical application (4^th^ bar) or oral gavage (5^th^ bar) significantly inhibited (38% to 46%, *P* < 0.001) UVB-induced suppression of CHS in mice compared with the mice that were not treated with honokiol but exposed to UVB radiation (Fig. 6a). Importantly, the level of inhibition of CHS was not significantly different between the two modes of administration of honokiol (Fig. 6a).

### Comparison of effects of honokiol with commercially available anti-cancer drugs on UVB-induced immunosuppression

Finally, we compared the effect of honokiol on UVB-induced immunosuppression with two cancer drugs which are used in the treatment of skin cancer, imiquimod (IMQ) and 5-flurouracil (FU)^23^. The CHS response was measured in C3H/HeN mice after topical treatment with equimolar concentrations (18.8 mM) of honokiol, IMQ, or 5-FU. As shown in Fig. 6b, topical treatment with each of these three agents significantly inhibited UVB-induced suppression of the CHS response (58% to 69%, *P* < 0.001) in mice. The percentages of inhibition of UVB-induced suppression of CHS by honokiol, IMQ or 5-FU were similar (4^th^, 5^th^ and 6^th^ bar) and there were no significant differences in the CHS responses among the agents tested.

## Discussion

UV radiation exposure induces inflammation and mediators of inflammation have been implicated in the initiation and development of several skin diseases, including melanoma and non-melanoma skin cancers^3^. UVB induction of the PG metabolite, PGE_2_, plays a major role in suppression of the immune system, and several lines of evidence suggest that UVB-induced immunosuppression is a risk factor for skin malignancy^3, 8^. By employing different experimental approaches, we have shown previously that UVB-induced suppression of CHS is associated with the overexpression of COX-2 and PGE_2_^8, 14^. In our prior studies, we also established a link between UVB-induced inflammation and UVB-induced DNA hypermethylation in UVB-exposed skin^12–14^. That is, UVB-induced inflammation initiates or mediates DNA hypermethylation and this DNA hypermethylation has a role in UVB-induced suppression of CHS response. As we had shown that topical application of honokiol inhibits UVB radiation-induced skin tumor development in mice, we sought to determine whether inhibition of skin carcinogenesis by honokiol is due to the inhibition of UVB-induced immunosuppression. We therefore tested the effects of honokiol on UVB-induced inflammation using the CHS model and further tested whether COX-2, PGE_2_ and DNA hypermethylation are molecular targets in this model. In these experiments, we used a hydrophilic cream-based topical formulation of honokiol that we have developed that can be used safely and easily^15^. The current study clearly reveals that topical application of this formulation of honokiol significantly inhibits UVB radiation-induced suppression of CHS response in mice, and that this suppression is associated with inhibition of UVB-induced inflammatory mediators, including COX-2 overexpression, PGE_2_ production and downregulation of PGE_2_ receptors. The current study also suggests that the inhibitory effects of topical application of honokiol on UVB-induced immunosuppression persist for some time after the original application. It has been shown in previous studies that UVB-induced inflammation incurs epigenetic alterations in the mouse skin, including enhancement of DNA methylation and stimulation of Dnmt activity^12–14^. Our current studies demonstrate that honokiol does not inhibit UVB-induced suppression of the CHS response in COX-2-deficient mice although it inhibits UVB-induced suppression of the CHS response in their wild-type littermates. Moreover, treatment of UVB-exposed COX-2-deficient mice with PGE_2_ reinstated suppression of the CHS response and topical application of honokiol inhibited this PGE_2_-mediated suppression of CHS in UVB-irradiated COX-2-deficient mice.

Previous studies of COX-2 proficient and COX-2-deficient mice have shown a link between UVB-induced overexpression of PGE_2_ and DNA hypermethylation in UVB-irradiated mouse skin^13, 14^. NSAIDs and demethylating agents also have been used in mouse models to further establish a link between inflammation and DNA hypermethylation and their involvement in immunosuppression in UVB-exposed mice. DNA hypermethylation has been shown to affect numerous genes thereby initiating or exacerbating cancer driving events. We therefore determined the effects of honokiol on the DNA hypermethylation in UVB- exposed mouse skin. The results demonstrate that topical application of honokiol inhibits or blocks UVB-induced DNA hypermethylation as well as Dnmt activity in UVB-exposed mouse skin. Furthermore, honokiol inhibits the transcription regulators of Dnmt activity, Sp1 and Sp3, which have been implicated in DNA demethylation. In addition to blocking the addition of extra methyl groups to the 5^th^ position of cytosine through inhibiting Dnmt activity, honokiol treatment also promotes DNA demethylation of existing DNA hypermethylation through activation of the TET enzyme and expression of TET proteins. Active DNA demethylation by TET proteins has been shown to play critical roles in T cell functions and particularly cytokine expression^24^. This is consistent with the literature indicating that epigenetic modifications in general, and especially aberrant DNA methylation, play important role in the regulation of cytokines in malignancies^25–28^. These studies help us to understand the mechanisms and modes of action through which honokiol inhibits UVB-induced immunosuppression. In addition to the role of inflammation in DNA hypermethylation and DNMT activity, oxidative stress also has been shown to play a role in stimulation of hypermethylation, thus it is likely that UVB induced reactive oxygen species are mediating the increase in DNMTs and that leads to hypermethylation^29–31^. Honokiol treatment also has been shown to increase the mitochondrial rate of oxygen consumption and reduces reactive oxygen species synthesis and activates mitochondrial enzyme Sirt3. These changes deacetylates MnSOD, thus activating this enzyme and also affects the cardiac hypertrophy in mice^32, 33^.

**Figure 7:**
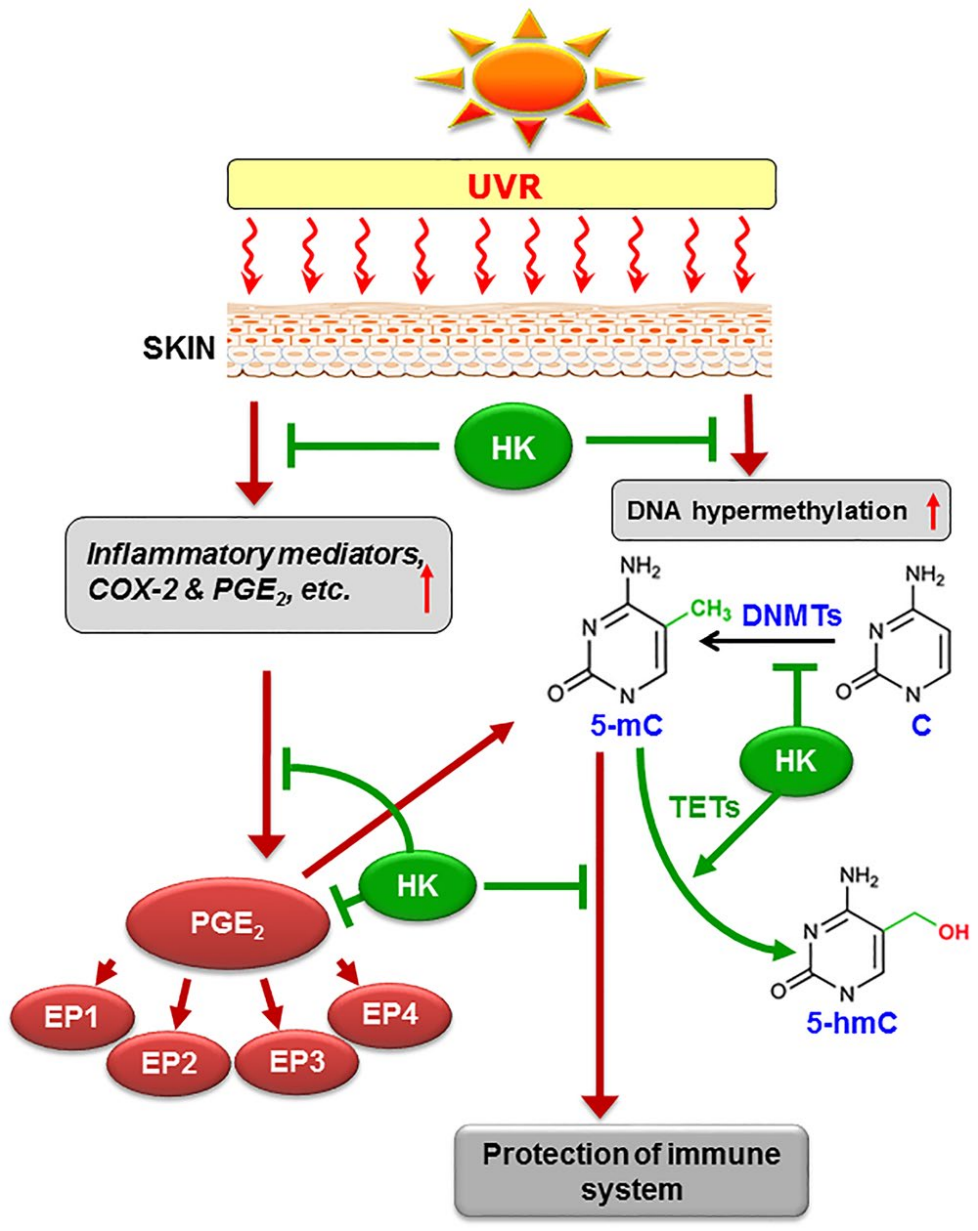
Schematic diagram depicts a crosstalk between UV radiation-induced inflammatory mediators and epigenetic regulators (DNA hypermethylation). UVB-induced photodamage initiates the generation of inflammatory mediators and enhancement in DNA methylation in skin cells. Inflammatory mediators and DNA hypermethylation play critical roles in suppression of immune system in UV-exposed mice. Topical application of honokiol inhibits UVB-induced inflammation as well as DNA hypermethylation through TET-mediated demethylation of DNA, and that results in restoration or protection of immune system in UVB-exposed mouse skin. HK, honokiol; 5mC, 5-methylcytosine; 5-hmC, 5-hydroxymethylcytosine; TET, ten eleven translocation.

The systemic anti-cancer chemotherapies that currently are approved for the treatment of skin cancers can cause side-effects, including life-threatining suppression of the immune system, and resistance to the therapies can develop on long-term use. In our experimental setting, we found that the chemopreventive effects of topical application of honokiol were similar to IMQ and 5-FU in terms of inhibition of UVB-induced immunosuppression. Although, immunotherapy and targeted therapies have recently been introduced for treatment of skin cancers, including advanced, metastatic disease, these approaches are not readily translatable to the consistent, long-term administration required for effective prevention. Moreover, the current experimental findings advance our understanding of UVB-induced immunosuppression and provide clues for development of new chemopreventive strategies through blocking of the induction of inflammatory axis and epigenetic modifications, specifically DNA hypermethylation in UVB-exposed skin, as summarized in Fig. 7. Taken together with the observation that adverse effects have not been observed during the experiments investigating topical application of honokiol, these data indicate that clinical trials of honokiol should be considered to determine whether it is an effective alternative strategy to control cutaneous malignancies. It is important to continue these investigations because skin cancer is among the most costly of all cancers to treat^34^.

## Materials and Methods

### Antibodies and reagents

Antibodies specific for EP2, EP3, EP4, Sp1, Sp3, TET2, TET3, β-actin, vinculin, and histone H3 were purchased from Santa Cruz Biotechnology (Dallas, TX). The following antibodies were purchased: COX-2 and 5-methylcytosine (5mC) from Cell Signaling (Danvers, MA); EP1 from Abcam (Cambridge, MA); Dnmt1, Dnmt3a, and Dnmt3b from Novus Biologicals (Littleton, CO); and TET1 from Epigentek, Inc (New York, NY). The Methylamp Global DNA Methylation Quantification Kit, the EpiQuik DNA Methyltransferase Activity Assay Kit, and TET Assay Activity Kit (Epigenase 5mC-hydroxylase TET activity/inhibition assay kit) were purchased from Epigentek, Inc. (New York, NY). The PGE_2_ immunoassay kit was purchased from Cayman Chemical (Ann Arbor, MI). All other chemicals of analytical grade were purchased from Sigma-Aldrich Chemical Co (St Louis, MO). Purified honokiol was purchased from Quality Phytochemicals, LLC (Edison, NJ).

### Animals

Female C3H/HeN mice (5–6 weeks old) were purchased from Charles River Laboratory (Wilmington, MA). The breeding pairs of COX-2 deficient (+/−) mice on the 129 Ola/C57BL/6 background were kindly provided by Dr. Langenbach, National Institutes of Environmental Health Sciences (National Institutes of Health). The COX-2 deficient mice used in this study were bred using heterozygous male and female pairs in our animal resource facility as described^35^. The wild-type littermates also were used in this study. The health status of the COX-2-deficient mice was normal as compared to their wild-type littermates, and they did not show any gross phenotypic differences. All experimental animals were maintained under standard housing conditions of 12-h dark/12-h light cycle, a temperature of 24 ± 2 °C, and relative humidity of 50 ± 10%. Food and water were provided to the animals *ad libitum*. The animal protocol for this study was approved by the Institutional Animal Care and Use Committee (IACUC) of the University of Alabama at Birmingham. Mice were housed in the Animal Resource Facility and all methods were performed in accordance with the guidelines and regulations of IACUC.

### UVB irradiation of mice

The dorsal hair of the mice was shaved using electric clippers at least 24 h before UVB exposure. The shaved dorsal skin of the mice was exposed to UVB radiation as described earlier^36, 37^ using a band of four FS20 UVB lamps (Daavlin, UVA/UVB Research Irradiation Unit, Bryan, OH) equipped with an electronic controller to regulate UV dosage. The UV lamps emit UVB (280–320 nm; ≈80% of total energy) with UVC emission being insignificant. The peak emission of UV radiation is at 314 nm. This equipment enables us to enter the UV dose in millijoules/cm^2^ and variations in energy output are compensated automatically so that the desired UV dose is delivered at the skin site.

### Contact hypersensitivity (CHS) model for assessment of immunosuppression

The UVB radiation-induced suppression of the immune system in mice was assessed using the CHS model, as described previously^8, 37^. This protocol is commonly used for this purpose. Briefly, the clipper-shaved dorsal skin of the mice was exposed to UVB radiation (150 mJ/cm^2^) on four consecutive days. During the exposure of the mice to the UV radiation, the ears of the mice were protected from the UV irradiation. Twenty-four hours after the last UV exposure, the mice were sensitized by applying 25 µl of 0.5% DNFB in an acetone: olive oil mixture (4:1, v/v) on the UVB-exposed site. The CHS response was elicited 5 days later by treating both surfaces of both the ears of each mouse with 20 µl of 0.2% DNFB in the acetone: olive oil mixture (4:1, v/v). The ear skin thickness was measured 24 h after the challenge using an engineer’s micrometer (Mitutoyo, Tokyo, Japan) and was compared with the ear thickness just before the challenge, as described earlier^8, 36, 37^. Animals that were not exposed to UV irradiation but were sensitized and challenged served as a positive control. Mice that were not UV irradiated and received only ear challenge without sensitization with DNFB served as a negative control. Each treatment group consisted of four to five mice. The UV-induced suppression of CHS response was calculated as detailed previously^36, 37^.

### Protocols for testing the effects of treatment of mice with honokiol, indomethacin, 5-Aza-dc, PGE_2_ and cancer drugs on the CHS responses

To assess the effects of topical application of honokiol on UVB-induced suppression of the CHS response, honokiol (0.5 and 1.0 mg/cm^2^ of skin area) in a hydrophilic cream-based topical formulation was applied to the clipper-shaved mouse skin starting 3 days before the beginning of UVB exposure and then 30 min before each UVB exposure. Briefly, honokiol was topically applied at the dose of 0.5 and 1.0 mg/cm^2^ skin area after mixing it in hydrophilic ointment base. Approximately 4 cm^2^ skin area was covered for topical application on each mouse. 50 mg/mouse topical formulation was used. It means 2 mg and 4 mg honokiol was used per mouse/50 mg ointment base. In other words, honokiol was used at the dose of 4% and 8% (w/w) in topical formulation. We have used hydrophilic ointment base as a vehicle for this topical formulation. This vehicle or hydrophilic ointment is available over-the-counter and named as “AquaBASE Ointment”, manufactured by Perrigo (Minneapolis, MN). In other specified experiments, honokiol was administered to the mice by oral gavage in PBS (2 mg/mouse/0.2 ml) 30 min before each UVB exposure. The effects of indomethacin were tested by topical application of indomethacin (50 µg) in 0.2 mL acetone 30 min before each UVB exposure. PGE_2_ (50 µg in 0.2 mL acetone) was topically applied to the COX-2-deficient mouse skin after each UVB exposure^38, 39^. To verify the role of UVB-induced DNA hypermethylation in UVB-induced immunosuppression, mice were administered the DNA demethylating agent (5-Aza-dc; 1 mg/kg body weight, *i.p*.) after exposure to UVB radiation^40^. The experimental mice received two doses of 5-Aza-dc. The first dose was injected after the first UVB exposure, while the second dose was injected after the third exposure to UVB radiation as we have described in previous studies^8^. To compare the effect of honokiol with imiquimod and 5-fluorouracil, the mice were topically administered imiquimod and 5-fluorouracil in equimolar concentrations (18.8 mM), which is equivalent to the dose of honokiol (1.0 mg/cm^2^ of skin area). The topical application was initiated 3 days before the beginning of UVB irradiation (once in a day) and during the UVB irradiation protocol.

### Immunoassays for PGE_2_

Skin tissues obtained from mice were homogenized in 100 mM phosphate buffer (pH 7.4) containing 1 mM EDTA and 10 μM indomethacin using a Polytron homogenizer (Fisher Scientific, Pittsburg, PA) on ice. The clarified supernatants were used to measure the levels of PGE_2_ using the Cayman PGE_2_ Enzyme Immunoassay Kit (Cayman Chemical) following the manufacturer’s instructions.

### Analysis of 5-mC using dot-blot analysis

To determine the levels of DNA methylation in the skin tissues, total genomic DNA was isolated and dot-blot analysis performed as described previously^36, 41^. Briefly, genomic DNA (50 ng) was transferred to a positively charged nitrocellulose membrane using a vacuum dot-blot apparatus (Bio-Dot Apparatus; Bio-Rad, Hercules, CA). After blocking the nonspecific binding sites by immersion in blocking buffer, the membrane was incubated with the antibodies specific for 5-mC at room temperature. After washing, the membrane was incubated with HRP-conjugated secondary antibody. The circular bands of 5-mC were detected using enhanced chemiluminescence reagents.

### Analysis of global DNA methylation

The levels of global DNA methylation in the isolated skin samples were determined by extraction of total genomic DNA followed by analysis using the Methylamp Global DNA Methylation Quantification Kit (Epigentek, Inc) following the manufacturer’s protocol.

### Assays of Dnmt and TET activity

The skin tissues from different treatment groups were used to prepare nuclear extracts using the EpiQuik Nuclear Extraction Kit (Epigentek, Inc). Dnmt activity in nuclear extracts was determined using the EpiQuik DNA Methyltransferase Activity Assay Kit (Epigentek, Inc.) and TET Activity was determined using Epigenase 5mC-hydroxylase TET Activity/Inhibition Assay Kit (Epigentek, Inc.) following the instructions provided by the manufacturer.

### Western blot analysis

The cytosolic and nuclear fractions of skin tissues were prepared and subjected to the analysis of different protein biomarkers using western blot analysis, as described previously^41, 42^. Proteins (60–80 μg) were resolved on 4–15% gradient gels (Mini-Protean TGX precast gels) purchased from Bio-Rad (Hercules, CA). After transfer and blocking of the non-specific sites, the nitrocellulose membranes were incubated with the primary antibodies in blocking buffer overnight at 4 °C. The membrane was then washed with Tris-buffered saline, 0.1% Tween 20 and incubated with HRP-conjugated secondary antibody. Protein bands were visualized using the enhanced chemiluminescence detection reagents. Equal loading of proteins on the gel was verified by re-probing the membrane with anti-β-actin, anti-histone H3, or anti-vinculin antibodies. Immunoblot data are presented in duplicate from each treatment group in which each lysate sample was prepared by pooling the skin samples from 2 or 3 different mice, and samples were run simultaneously on the same gel. By doing this, we can compare the data from different mice.

### Immunofluorescent detection of 5-mC-positive cells in mouse skin

Skin biopsies from the mice of the different treatment groups were collected and frozen in OCT medium. For immunofluorescence detection of 5-mC-positive (DNA-methylated cells) cells, 5 μm thick frozen sections were thawed at room temperature in the dark and immersed in 70 mM NaOH solution in 70% ethanol for 2 min to denature nuclear DNA followed by neutralization in 100 mM Tris-HCl (pH 7.5) in 70% ethanol. The sections were then washed and permeabilized with 0.2% Triton-X100 in PBS for 20 min. After blocking with 5% bovine serum albumin, sections were incubated with antibody specific for 5-mC at 4 °C overnight. Sections were then washed and incubated with fluorescence-conjugated secondary goat anti-rabbit IgG and streptavidin Alexa fluor594 antibody. Sections were mounted with Vectashield mounting medium for fluorescence and stained with 4′, 6-diamidino-2-phenylindole (DAPI). Sections were examined using an Olympus Microscope (BX41, Tokyo, Japan) equipped for fluorescence analysis and with a Q color5 camera and CellSens software.

### Histological analysis

Skin tissues were collected from the ears of mice of each group 24 h after DNFB challenge, fixed in 10% formalin and paraffin blocks prepared. The 5 μm thick sections were subjected to standard H&E staining and photomicrographs obtained for analysis.

### Statistical analysis

The statistical significance of difference between the values of control and treatment groups was determined using analysis of variance, and using GraphPad Software, University of California, San Diego, CA. In each case, *P* < 0.05 was considered as statistically significant.

## References

[1] Katiyar SK (2007). UV-induced immune suppression and photocarcinogenesis: Chemoprevention by dietary botanical agents. Cancer Letts..

[2] Katiyar S, Elmets CA, Katiyar SK (2007). Green Tea and Skin Cancer: Photoimmunology, angiogenesis and DNA repair. J. Nutr. Biochem..

[3] Mukhtar H, Elmets CA (1996). Photocarcinogenesis: mechanisms, models and human health implications. Photochem. Photobiol..

[4] Kinlen L, Sheil A, Peta J, Doll R (1979). Collaborative United Kingdom-Australia study of cancer in patients treated with immunosuppressive drugs. Br. J. Med..

[5] Ondrus D (1999). The incidence of tumors in renal transplant recipients with long-term immunosuppressive therapy. Int. Urol. Nephrol..

[6] Yoshikawa T (1990). Susceptibility to effects of UVB radiation on induction of contact hypersensitivity as a risk factor for skin cancer in humans. J. Invest. Dermatol..

[7] Meunier L, Raison-Peyron N, Meynadier J (1998). UV-induced immunosuppression and skin cancers. Rev. Med. Interne.

[8] Prasad R, Katiyar SK (2013). Prostaglandin E2 promotes UV radiation-induced immune suppression through DNA hypermethylation. Neoplasia.

[9] Chung HT, Burnham DK, Robertson B, Roberts LK, Daynes RA (1986). Involvement of prostaglandins in the immune alterations caused by the exposure of mice to ultraviolet radiation. J. Immunol..

[10] Hart PH, Townley SL, Grimbaldeston MA, Khalil Z, Finlay-Jones JJ (2002). Mast cells, neuropeptides, histamine, and prostaglandins in UV-induced systemic immunosuppression. Methods.

[11] Elmets CA (2010). Chemoprevention of nonmelanoma skin cancer with celecoxib: a randomized, double-blind, placebo-controlled trial. J. Natl. Cancer Inst..

[12] Nandakumar V, Vaid M, Tollefsbol TO, Katiyar SK (2011). Aberrant DNA hypermethylation patterns lead to transcriptional silencing of tumor suppressor genes in UVB-exposed skin and UVB-induced skin tumors of mice. Carcinogenesis.

[13] Katiyar SK, Singh T, Prasad R, Sun Q, Vaid M (2012). Epigenetic alterations in ultraviolet radiation-induced skin carcinogenesis: Interaction of bioactive dietary components on epigenetic targets. Photochem. Photobiol..

[14] Prasad, R. & Katiyar, S. K. Crosstalk among UV-induced inflammatory mediators, DNA damage and epigenetic regulators facilitates suppression of the immune system. *Photochem. Photobiol*. doi:10.1111/php.12687 (2016). 10.1111/php.12687 PMC5466507 27935057

[15] Vaid M, Sharma SD, Katiyar SK (2010). Honokiol, a phytochemical from the Magnolia plant, inhibits photocarcinogenesis by targeting UVB-induced inflammatory mediators and cell cycle regulators: development of topical formulation. Carcinogenesis.

[16] Chilampalli S (2010). Chemopreventive effects of honokiol on UVB-induced skin cancer development. Anticancer Res..

[17] Prasad R, Katiyar SK (2016). Honokiol, an active compound of Magnolia plant, inhibits growth, and progression of cancers of different organs. Adv. Exp. Med. Biol..

[18] Munroe ME, Arbiser JL, Bishop GA (2007). Honokiol, a natural plant product, inhibits inflammatory signals and alleviates inflammatory arthritis. J. Immunol..

[19] Elmets CA, Bergstresser PR, Tigelaar RE, Wood PJ, Streilein JW (1983). Analysis of the mechanism of unresponsiveness produced by haptens painted on skin exposed to low dose ultraviolet radiation. J. Exp. Med..

[20] Jinawath A, Miyake S, Yanagisawa Y, Akiyama Y, Yuasa Y (2005). Transcriptional regulation of the human DNA methyltransferase 3A and 3B genes by Sp3 and Sp1 zinc finger proteins. Biochem. J..

[21] Kishikawa S, Murata T, Kimura H, Shiota K, Yokoyama KK (2002). Regulation of transcription of the Dnmt1 gene by Sp1 and Sp3 zinc finger proteins. Eur. J. Biochem..

[22] Rasmussen KD, Helin K (2016). Role of TET enzymes in DNA methylation, development, and cancer. Genes Dev..

[23] https://www.cancer.gov/about-cancer/treatment/drugs/skin. National cancer institute website, accessed on Nov 29 (2016).

[24] Ichiyama K (2015). The methylcytosine dioxygenase Tet2 promotes DNA demethylation and activation of cytokine gene expression in T cells. Immunity.

[25] Cheng C (2014). SOCS1 hypermethylation mediated by DNMT1 is associated with lipopolysaccharide induced inflammatory cytokines in macrophages. Toxicol. Lett..

[26] Rosenzweig JM, Glenn JD, Calabresi PA, Whartenby KA (2013). KLF4 modulates expression of IL-6 in dendritic cells via both promoter activation and epigenetic modification. J. Biol. Chem..

[27] Tekpli X (2013). DNA methylation at promoter regions of interleukin 1B, interleukin 6, and interleukin 8 in non-small cell lung cancer. Cancer Immunol. Immunother..

[28] Mocellin S, Wang E, Marincola FM (2001). Cytokines and immune response in the tumor microenvironment. J. Immunother..

[29] Govindarajan B (2002). Reactive oxygen-induced carcinogenesis causes hypermethylation of p16(Ink4a) and activation of MAP kinase. Mol. Med..

[30] Arbiser JL (2006). Presence of p16 hypermethylation and Epstein-Barr virus infection in transplant-associated hematolymphoid neoplasm of the skin. J. Am. Acad. Dermatol..

[31] Bonner MY, Arbiser JL (2012). Targeting NADPH oxidases for the treatment of cancer and inflammation. Cell. Mol. Life Sci..

[32] Akamata K (2016). SIRT3 is attenuated in systemic sclerosis skin and lungs, and its pharmacologic activation mitigates organ fibrosis. Oncotarget..

[33] Pillai VB (2015). Honokiol blocks and reverses cardiac hypertrophy in mice by activating mitochondrial Sirt3. Nat. Commun..

[34] Housman TS (2003). Skin cancer is among the most costly of all cancers to treat for the Medicare population. J. Am. Acad. Dermatol..

[35] Langenbach R, Loftin C, Lee C, Tiano H (1999). Cyclooxygenase knockout mice: models for elucidating isoform-specific functions. Biochem. Pharmacol..

[36] Katiyar SK, Vaid M, Steeg HV, Meeran SM (2010). Green tea polyphenols prevent UV-induced immunosuppression by rapid repair of DNA damage and enhancement of nucleotide excision repair genes. Cancer Prev. Res..

[37] Meeran SM, Katiyar S, Elmets CA, Katiyar SK (2006). Silymarin inhibits UV radiation-induced immunosuppression through augmentation of interleukin-12 in mice. Mol. Cancer Ther..

[38] Chun KS, Langenbach R (2011). The prostaglandin E2 receptor, EP2, regulates survivin expression via an EGFR/STAT3 pathway in UVB-exposed mouse skin. Mol. Carcinog..

[39] Chun KS, Akunda JK, Langenbach R (2007). Cyclooxygenase-2 inhibits UVB-induced apoptosis in mouse skin by activating the prostaglandin E2 receptors, EP2 and EP4. Cancer Res..

[40] Xia D, Wang D, Kim SH, Katoh H, DuBois RN (2012). Prostaglandin E2 promotes intestinal tumor growth via DNA methylation. Nat. Med..

[41] Vaid M, Prasad R, Singh T, Jones V, Katiyar SK (2012). Grape seed proanthocyanidins reactivate silenced tumor suppressor genes in human skin cancer cells by targeting epigenetic regulators. Toxicol. Appl. Pharmacol..

[42] Mantena SK, Sharma SD, Katiyar SK (2006). Berberine, a natural product, induces G1 phase cell cycle arrest and caspase-3-dependent apoptosis in human prostate carcinoma cells. Mol. Cancer Ther..

